# The role of *Lactobacillus plantarum* (camel milk isolate) in inflammation and acute pain modulation: a comparative study with acetaminophen in animal model

**DOI:** 10.1097/MS9.0000000000005398

**Published:** 2026-07-27

**Authors:** Shahdad Ahmadi, Negin Khosravipour, Sara Pedram-Jafari, Somayeh Dashti, Soroush Taherkhani, Leila Roozbeh Nasiraie, Atousa Janzadeh

**Affiliations:** a Neuromusculoskeletal Research Center, Iran University of Medical Sciences, Tehran, Iran; b Physiology Research Center, Iran University of Medical Sciences, Tehran, Iran; c Cellular and Molecular Research Center, Iran university of Medical Sciences, Tehran, Iran; d Department of Food Science and Technology, No.C., Islamic Azad University, Noor, Iran

**Keywords:** acute pain, gut-brain axis, inflammation, Lactobacillus plantarum, probiotic

## Abstract

**Background:**

Recent studies indicate that modulation of the gut-microbiota through probiotics may offer innovative therapeutic alternatives. This investigation assessed the analgesic and anti-inflammatory effects of *Lactobacillus plantarum* NIMBB003, a probiotic strain isolated from camel milk, using a rat model of acute inflammatory pain.

**Methods:**

Forty-two male Wistar rats were divided into seven groups, including controls, formalin-induced pain, formalin with vehicle, formalin with acetaminophen, and three groups receiving oral *L. plantarum* at doses of 10^7^, 10^9^, and 10^11^ CFU/mL for 14 days. Acute pain was induced via the formalin test, assessing nociceptive behaviors during early (0–5 minutes) and late (15–60 minutes) phases. Serum inflammatory cytokines interleukin (IL)-1β, IL-2, and IL-6 were measured.

**Results:**

In this study, the highest probiotic dose (10^11^ CFU/mL) produced significant analgesia of a similar magnitude to acetaminophen, with reduced pain behaviors in both phases of the formalin test (*P* < 0.001). Lower probiotic doses did not yield significant analgesic effects. Moreover, high-dose *L. plantarum* and acetaminophen treatments significantly lowered serum levels of pro-inflammatory cytokines IL-1β, IL-2, and IL-6 (*P* < 0.05 to *P* < 0.01).

**Conclusion:**

These results demonstrate that *L. plantarum* NIMBB003 has strong, dose-dependent analgesic and anti-inflammatory effects in an acute pain model. At a concentration of 10^11^ CFU/mL, its efficacy was in the same direction as that of acetaminophen in this model, suggesting its potential as a safe and effective alternative for treating acute inflammatory pain. This is consistent with increasing evidence that probiotics can influence behavioral antinociception with minimal impact on systemic inflammation.

## Graphical abstract

Graphical abstract available at: https://links.lww.com/MS9/B314

**Figure:**
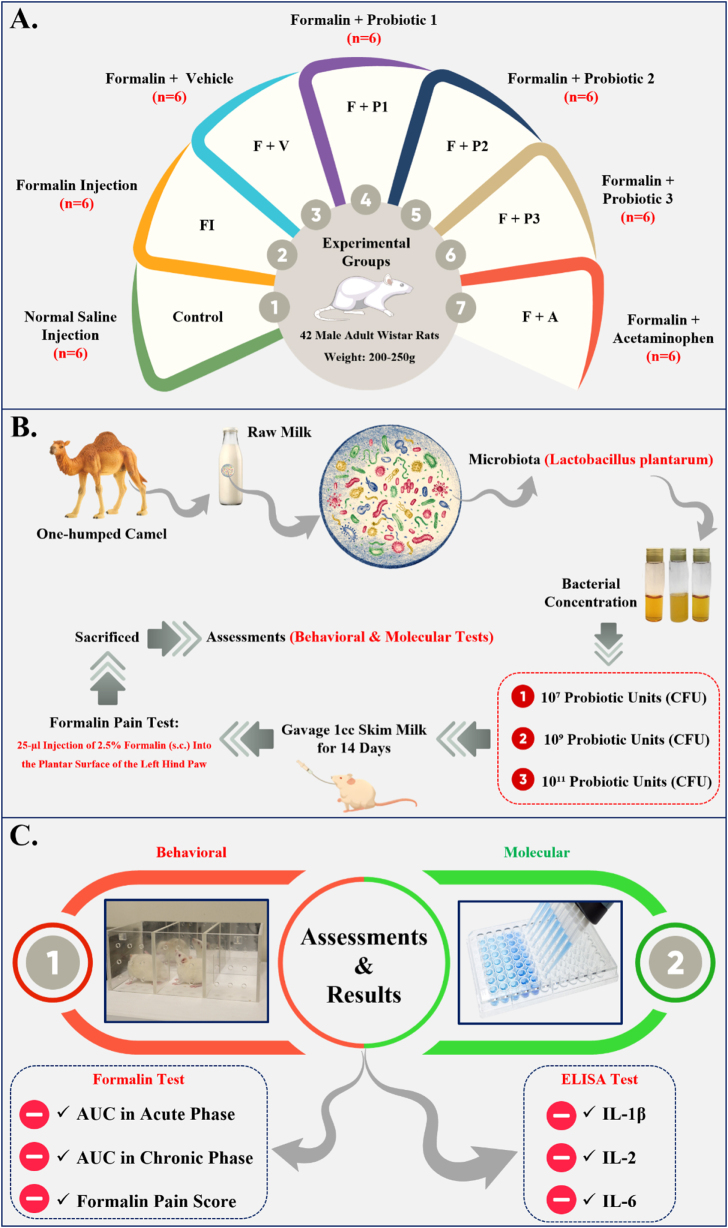

## Introduction

**Figure 1.**
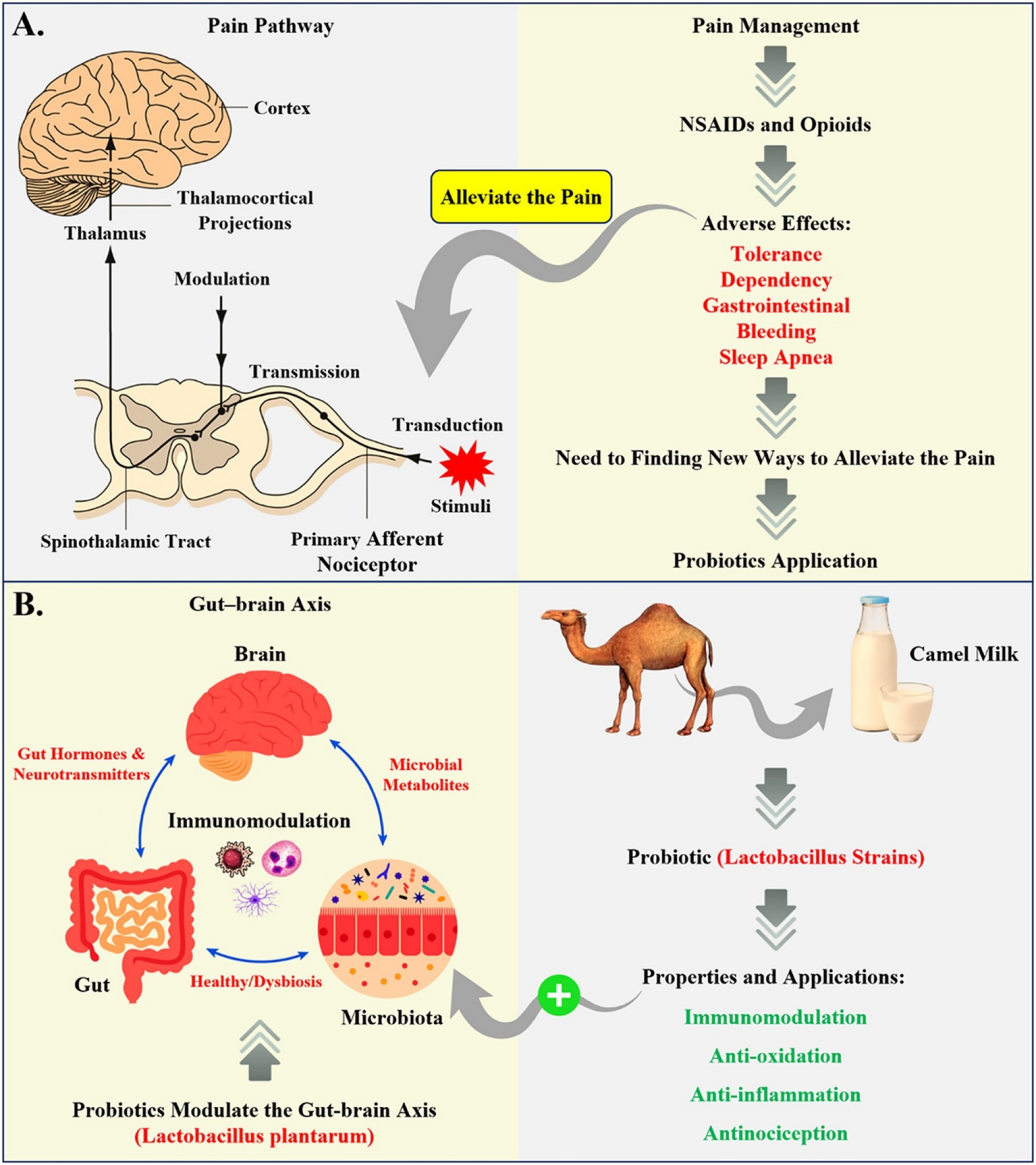
Among the available pain management options is the use of NSAIDs and opioids, which have been demonstrated to reduce pain by affecting the pain nerve signaling pathway. However, due to their interactions with other physiological systems, these drugs can also result in various adverse effects. Consequently, the utilization of alternative therapeutic agents, such as probiotics, which are associated with a reduced incidence of adverse effects, may prove advantageous (A). Probiotics derived from camel milk have potential benefits for the gut-brain axis, a bidirectional communication system between the gastrointestinal tract and the central nervous system. Camel milk itself is rich in bioactive compounds, and when fermented with probiotics (such as *Lactobacillus* strains), it may enhance gut and brain health as well as normal functions, including immunomodulatory effects (B).

Acute pain, which is an abrupt onset of discomfort with a specific cause, has a substantial impact on patients’ quality of life and places a heavy burden on healthcare systems around the world. Although NSAIDs and opioids are the cornerstones of analgesic therapy, significant adverse effects, such as tolerance, dependency, gastrointestinal bleeding, or even sleep apnea, limit their long-term use^[^1^]^. Because of these side effects, there is a growing interest in finding new ways to alleviate the pain of patients. A particularly promising field of study is the gut–brain axis, a bidirectional communication link between the central nervous system and the gut microbiota. Recent studies have shown that gut microbiota may play a significant role in regulating pain perception by influencing nociception and inflammation through immunological, endocrine, and neurological pathways^[^2,3^]^ (Fig. 1A).

Probiotics are live microorganisms that, when taken in the right amounts, can actually support their host’s health. Beyond their well-known roles in gut health and regulating the immune system, they can also influence neuroimmune interactions^[^4^]^.

In animal studies, certain probiotic strains have shown promise for easing pain and dampening inflammation. For example, in rodent models of neuropathy, Lactobacillus strains were found to reduce pain and shift macrophages toward an anti-inflammatory state^[^2,5^]^. That said, we still don’t fully understand how individual strains impact acute nociceptive pain, since most work so far has zeroed in on gastrointestinal or neuropathic pain. There’s a clear need for further studies to unravel the exact mechanisms, especially those involving immune modulation and microbial metabolites.

*Lactobacillus plantarum* is one of the many probiotic species that is well known for its immunomodulatory, antioxidant, and anti-inflammatory properties^[^5^]^. By modifying the production of short-chain fatty acids and cytokine signaling, Huang *et al* demonstrated that the oral administration of *L. plantarum* reduced neuropathic pain and preserved nerve integrity^[^2^]^. It is interesting to note that lactic acid bacteria with antimicrobial and anti-inflammatory properties have been found in abundance in camel milk^[^6,7^]^. Specifically, camel milk isolates of *L. plantarum* show strong antagonistic activity against harmful bacteria and could be useful in medicine^[^7^]^ (Fig. 1B).

Afshar *et al* demonstrated that *L. plantarum* NIMBB003 at 10^9^ CFU/mL exhibited strong protective effects against AFB1-induced genotoxicity. This underscores the importance of incorporating local, natural, and native compounds with antioxidant properties, such as probiotics, into macro-management policies aimed at the development of green strategies. These strategies are intended to facilitate the discovery and production of new medicines and functional foods that offer protective or therapeutic benefits against nutritional and endogenous DNA toxins^[^8^]^.

The present study utilizes an experimental animal model to assess the impact of the camel milk-derived strain of *L. plantarum* NIMBB003 on acute pain. It is hypothesized that this strain offers a novel probiotic-based intervention for safer and more efficient pain management by reducing acute pain through anti-inflammatory or neuromodulatory pathways.

## Materials and methods

### Animals

Forty-two adult male Wistar rats were acquired from the Center for Experimental and Comparative Studies at Iran University of Medical Sciences. Upon arrival, the rats were randomly assigned to seven groups, and every four animals were housed in separate cages to acclimate to their new environment. Throughout the study, they had unrestricted access to food and water, and a controlled 12-hour light/dark cycle was maintained to ensure consistent living conditions.

**Figure 2.**
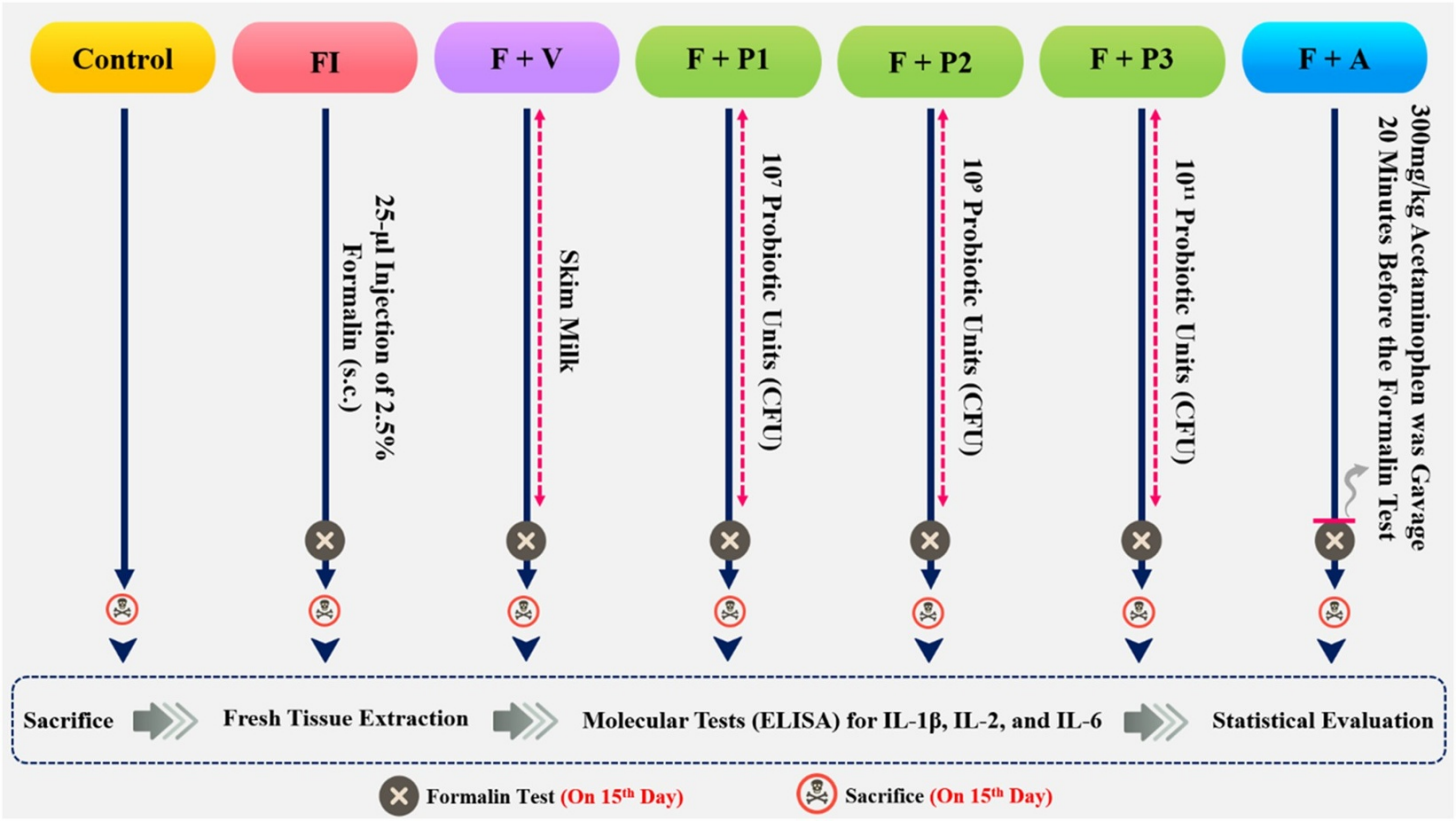
The experimental timeline protocols.

The sample size was determined based on previous studies using the formalin-induced pain model in rats (*n* = 6 per group). Similar group sizes have been used in studies evaluating antinociceptive effects in Wistar rats^[^9,10^]^. Animals received, via oral gavage, 1 cc of saline, formalin, or formalin combined with one of several doses of probiotics for 14 days^[^2^]^, or a single dose of acetaminophen syrup on the formalin test day (Table 1; Fig. 2). Twenty minutes following the final gavage, a subcutaneous injection of formalin was administered into the plantar surface of the left hind paw.

**Table 1:** Animal grouping and its description.

Groups	Explanation
Control	25-µL injection of Normal saline (s.c.) into the plantar surface of the left hind paw
Formalin injection (FI)	25-µL injection of 2.5% formalin (s.c.) into the plantar surface of the left hind paw
Formalin + probiotic 1 (F + P1)	Animals received 10^7^ probiotic units (cfu) in 1 cc skim milk, via gavage for 14 days, 20 minutes prior to the formalin test.
Formalin + probiotic 2 (F + P2)	Animals received 10^9^ probiotic units (cfu) in 1 cc skim milk, via gavage for 14 days, 20 minutes prior to the formalin test.
Formalin + probiotic 3 (F + P3)	Animals received 10^11^ probiotic units (cfu) in 1 cc skim milk, via gavage for 14 days, 20 minutes prior to the formalin test.
Formalin + acetaminophen (F + A)	1 cc of acetaminophen syrup (300 mg/kg, single dose) was gavage to the animals 20 minutes before the formalin test
Formalin + vehicle (F + V)	The animals were gavage 1cc skim milk for 14 days 20 minutes before formalin test.

This methodology provided a stable environment for the animals, facilitating reliable data collection and analysis during the experimental procedures. Careful consideration of ethical standards and animal welfare was crucial in conducting this research, ensuring that all methods were in accordance with established guidelines for the humane treatment of laboratory animals, as approved by the Ministry of Health and the Center for Experimental Studies of the University of Medical Sciences.

HIGHLIGHTS

- The administration of *Lactobacillus plantarum* NIMBB003 effectively reduced acute inflammatory pain in a dose-responsive fashion.
- *L. plantarum* NIMBB003 dose (10^11^ CFU) demonstrated significant analgesic activity in both early and late phases of the formalin-induced pain model, and its efficacy was of similar magnitude in this model to that of the reference analgesic, acetaminophen.
- Pretreatment with the high-dose probiotic markedly reduced pro-inflammatory cytokines, specifically IL-1β, IL-2, and IL-6. This anti-inflammatory effect was similar to that achieved by acetaminophen.
- Traditional analgesics, such as NSAIDs and opioids, are associated with adverse events, and the extraction of a probiotic strain from camel milk to manage inflammatory pain appears to be a viable alternative.

The article complies with the TITAN Guidelines 2025 – Governing Declaration^[^11^]^.

### L. plantarum extraction and probiotic preparation

The *L.* plantarum NIMBB003 strain was sourced from the culture collection center of the Cellular Laboratory at Shams Bavarane Salamate Nour Consulting & Production Services company, Tehran, Iran. This strain represents a novel native probiotic, originally isolated from the raw milk microbiota of one-humped camels in Golestan Province, Iran. It has been registered under the accession number MT012188.1 as a unique sequence in the NCBI/BLAST database^[^8^]^.

To prepare the working culture, frozen stock was transferred into de Man, Rogosa and Sharpe broth and incubated anaerobically (5% CO₂) at 37 ± 0.5°C for 11 ± 2 hours without agitation, following the growth curve established for the isolate. The bacterial cells were then harvested by centrifugation at 1500g for 10 minutes at 4°C, washed twice with sterile Ringer’s solution (pH 7.3), and finally re-suspended in 10% skim milk. The bacterial concentration was adjusted to 10^7^, 10^9^, and 10^11^ CFU/mL for subsequent use.

### Formalin test

For the formalin pain test, a Plexiglass chamber (35 cm × 35 cm × 35 cm) was used, with a mirror positioned at a 45° angle beneath the chamber to facilitate a clear view of the rat’s paws. The animals were placed individually in the chamber and allowed to habituate for 30 minutes prior to the tests. Each rat received a 25-µL injection of 2.5% formalin (s.c.) into the plantar surface of the left hind paw using a 27-gauge needle^[^12^]^.

Nociceptive scores were determined by an examiner blinded to the treated groups for each 5-minute interval by measuring the time spent in each of four behavioral categories: 0, where the position and posture of the injected hind paw are indistinguishable from the other hind paw; 1, where the injected paw bears little or no weight; 2, where the injected paw is elevated and not in contact with any surface; and 3, where the injected paw is licked, bitten, or shaken. The nociceptive score for each 5-minute block was calculated using the formula: (t0 × 0) + (t1 × 1) + (t2 × 2) + (t3 × 3) / (t0 + t1 + t2 + t3)^[^13^]^, which results in a score ranging from 0 to 3^[^9^]^. The test was divided into Phase I (early phase), which encompassed the first 5 minutes after intraplantar formalin injection (FI), and Phase II (late phase), which was considered to be from 15 to 60 minutes post-injection.

### Animal sacrifice

Two hours post-FI, the animals were deeply anesthetized using intraperitoneal administration of ketamine (90 mg/kg) and xylazine (9 mg/kg). Once complete anesthesia was confirmed, 3 cc of blood was collected from the left ventricle of each animal, and then the animals were rapidly decapitated. This blood was then transferred to a microtube and centrifuged (Tajhis Teb, Iran) at 2000 rpm for 5 minutes to separate the serum from the blood cells. The serum was carefully separated and stored in a −80°C freezer for future analysis.

### Evaluation of inflammatory interleukin

To assess interleukin (IL) levels in the blood following the formalin test, specific Enzyme-Linked Immunosorbent Assay (ELISA) kits were used on three samples from each group. IL-1β (R&D Systems, Cat. No. RLB00, USA), IL-2 (R&D Systems, Cat. No. R2000, USA), and IL-6 (Cusabio, Cat. No. CSB-E04640r, USA) were measured by a researcher blinded to the groups.

The method commenced with coating a 96-well plate with capture antibodies specific to each IL, followed by overnight incubation. After the plate was washed, samples or standards were added, facilitating the binding of ILs to the antibodies. Subsequently, detection antibodies linked to an enzyme were introduced. After another wash, a substrate solution was added, resulting in a colorimetric reaction that correlated with the concentration of each IL. The optical density was then measured at 450 nm using a microplate reader, and concentrations were calculated based on standard curves derived from known concentrations.

### Statistical analysis

Statistical analysis was conducted using SPSS version 22 software, with a significance threshold set at *P* < 0.05. One-way analysis of variance (ANOVA) was employed to evaluate the ELISA results, which are presented as mean ± SEM (standard error of the mean). The average scores recorded in the first 5 minutes were designated as phase 1, while the area under the curve (AUC) of pain scores monitored from 15 to 60 minutes post-FI was classified as phase 2. The calculated and normalized AUC values (indicating percentage decrease) for all groups were analyzed using one-way ANOVA, followed by protected Tukey’s test to compare the AUC values of the vehicle control (skim milk) group across early and late phases for multiple comparisons. The percentage decrease in AUC was interpreted as drug-induced antinociception during both phases of the formalin test. Additionally, a two-way ANOVA for repeated measures over time, followed by Bonferroni’s test, was utilized to assess the impact of time and treatment on the “pain score values” across all groups.

## Results

### Effects of probiotics administration on formalin-induced pain behaviors during the early and late phases

The results are presented as AUC values for the experimental groups during the early and late phases, with the FI group set to zero. One-way ANOVA revealed significant differences in AUC values for pain scores among the experimental groups in both the early [*F* (5, 35) = 22.58, *P* < 0.001] and late phases [*F* (3, 35) = 42.57, *P* < 0.001].

**Figure 3.**
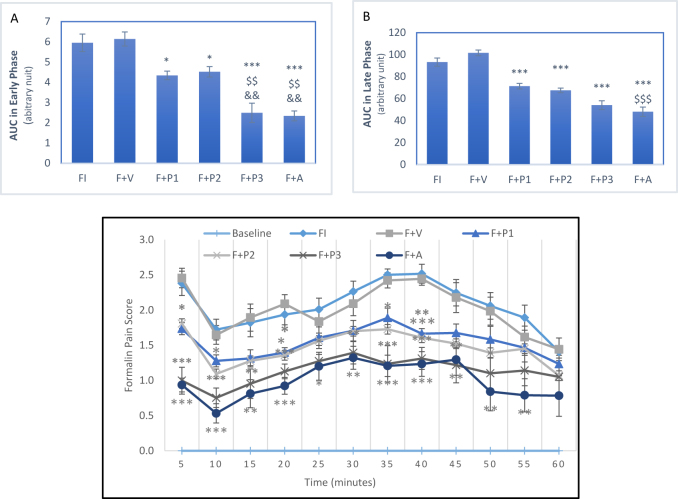
Comparison of the effects of acetaminophen and probiotic (Lactobacillus extracted from camel milk) on the response to formalin injection. Area under the curve (AUC) of pain scores during the early (A) and late (B) phases of the formalin test across different groups.

*Post hoc* Tukey’s test demonstrated a significant decrease in AUC values in all treated groups compared to the FI and F + V groups in both the early (*P* < 0.05 and *P* < 0.001) and late phases (*P* < 0.01 for both). Additionally, the results demonstrated a higher effect of the F + P3 group in reducing the AUC levels compared to the F + P1 and F + P2 groups in the early phase (*P* < 0.01 for both), as well as the F + A group compared to the F + P1 and F + P2 groups in both the early (*P* < 0.001 for both) and late phases (*P* < 0.001 for both) (Fig. 3A and B; Table 2).

* P < 0.05, *** P < 0.001 versus the FV group; $$ P < 0.01, $$$ P < 0.001 versus the F + P1 group; & P < 0.05, && P < 0.01, &&& P < 0.001 versus the F + P2 group. FI, formalin injection group; F + V, formalin + vehicle group; F + P1, formalin + probiotic 1 group; F + P2, formalin + probiotic 2 group; F + P3, formalin + probiotic 3 group; F + A, formalin + acetaminophen group. Data are presented as mean ± SEM (n = 6 per group).

**Table 2:** Mean differences of AUC values in early and late phases in the formalin pain test.

	Mean	*N*	SD	SEM
AUC_early phase
Formalin	5.95	6	1.06	0.43
FV	6.14	6	0.85	0.35
FP1	4.34	6	0.52	0.21
FP2	4.52	6	0.63	0.26
FP3	2.49	6	1.17	0.48
FA	2.34	6	0.60	0.24
AUC_late phase
Formalin	95.20	6	9.14	3.73
FV	101.62	6	6.41	2.62
FP1	71.31	6	6.10	2.49
FP2	67.49	6	5.04	2.06
FP3	54.03	6	9.86	4.03
FA	48.05	6	10.45	4.27

FI, formalin injection group; F + V, formalin + vehicle group; F + P1, formalin + probiotic 1 group; F + P2, formalin + probiotic 2 group; F + P3, formalin + probiotic 3 group; F + A, formalin + acetaminophen group.

In this experiment, treatment effects on pain scores over time were analyzed using two-way repeated measures ANOVA with Bonferroni’s *post hoc* test, which revealed significant main effects of time [*F* (11, 330) = 12.32, *P* < 0.001], while the treatment × time interaction effect was not significant [*F* (55, 330) = 0.74, *P* = 0.78] (sphericity assumed), indicating that treatment effects on pain scores remained consistent across time points. Between-subjects effects confirmed significant differences in pain scores between groups [*F* (5, 30) = 55.21, *P* < 0.001] (Fig. 3C).

### Effects of probiotic treatments on serum levels of inflammatory interleukins

#### IL-1β

**Figure 4.**
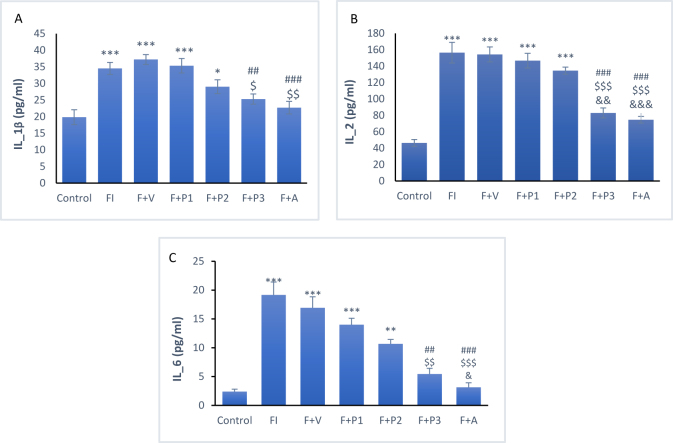
Results of serum IL-1β (A), IL-2 (B), and IL-6 (C) levels across different groups.

Analysis revealed significant differences in IL-1β concentrations across groups (F (6, 20) = 12.54, *P* < 0.001; *n* = 3/group). *Post hoc* Tukey tests identified substantially elevated IL-1β levels in the FI and F + V groups relative to controls (*P* < 0.001 for both). Treatments F + P1 and F + P2 showed no restorative effect on IL-1β levels compared to FI and F + V. In contrast, F + P3 significantly reduced IL-1β versus FI (*P* < 0.05), F + V (*P* < 0.01), and F + P1 (*P* < 0.001), while F + A achieved robust reductions against FI, F + V, and F + P1 (*P* < 0.01 for all) (Fig. 4A).

***P < 0.001, **P < 0.01, ***P < 0.001 versus Control; ##P < 0.01 and ###P < 0.001 versus F + V; $p < 0.05, $$P < 0.01, and $$$P < 0.001 versus F + P1; &P < 0.05, &&P < 0.01, and &&&P < 0.001 versus F + P2. FI, formalin injection group; F + V, formalin + vehicle group; F + P1, formalin + probiotic 1 group; F + P2, formalin + probiotic 2 group; F + P3, formalin + probiotic 3 group; F + A, formalin + acetaminophen group. Data are presented as mean ± SEM (n = 3 per group).

#### IL-2

Analysis of serum IL-2 levels revealed a significant difference between groups (*F* (6, 20) = 34.1, *P* < 0.001; *n* = 3 per group). *Post hoc* Tukey analysis demonstrated that the FI and F + V groups had significantly elevated IL-2 levels compared to the control group (*P* < 0.001 for both). Conversely, treatment with F + P3 and F + A effectively produced a reduction in IL-2 levels relative to F + V (*P* < 0.001 for both), as well as relative to F + P1 (*P* < 0.001 for both) and relative to F + P2 (*P* < 0.01, *P* < 0.01, respectively) (Fig. 4B).

#### IL-6

Analysis of serum IL-6 levels revealed a significant difference between groups (*F* = 6.20; *F* = 26.16, *P* < 0.001; *n* = 3 per group). *Post hoc* Tukey analysis showed that IL-6 levels were significantly higher in the FI and F-V groups compared to the control group (*P* < 0.001 for both).

Furthermore, the elevated IL-6 levels were significantly reduced by treatment with P2 (*P* < 0.01 and *P* < 0.05), P3, and F + A treatment (both *P* < 0.01), compared to the FI and F + V groups, respectively.

Furthermore, F + P3 and F + A treatments exhibited the strongest effect in restoring IL-6 levels compared to F + P1 (*P* < 0.01, *P* < 0.001, respectively), as well as F + A compared to F + P2 (*P* < 0.05) (Fig. 4C).

## Discussion

In this study, subcutaneous FI in animal footpads induced biphasic pain responses (early and late phases), consistent with prior research^[^14,15^]^. Elevated serum inflammatory IL (1β, 2, and 6) levels observed during the late phase align with mechanisms of central sensitization and inflammatory pathway activation. Spinal microglial activation, along with peripheral inflammation, may contribute to both the initiation and maintenance of central sensitization, leading to prolonged and lasting tactile hypersensitivity states after FI^[^16,17^]^.

Animals pretreated with probiotics prior to FI exhibited decreased pain responses during both the early and late phases, accompanied by a reduction in the expression of inflammatory ILs. However, this effect appears to be dose-dependent. The F *+* P2 and F *+* P1 groups showed no effect on either pain reduction or inflammatory ILs, while the F *+* P3 group’s pain decreased and reached levels similar to the control group.

Probiotics can more effectively shift cytokine profiles by increasing anti-inflammatory cytokines (e.g., IL-10) and reducing pro-inflammatory cytokines (e.g., tumor necrosis factor-alpha (TNF-α) and IL-6), which helps control inflammation and related pain^[^18^]^. Dose-dependent effects also relate to the ability of probiotics to modulate gut microbiota composition and barrier function, reducing endotoxin lipopolysaccharide translocation and systemic inflammation, which in turn alleviates inflammatory pain^[^19,20^]^. Insufficient doses may fail to reach the threshold needed to trigger these immune and metabolic pathways, while optimal doses maximize the therapeutic effects on both inflammation and pain^[^21^]^.

There are some controversies about the anti-inflammatory effects of probiotics. A meta-analysis by Wen Goh *et al* demonstrated that specific probiotic strains – including *L. fermentum* CECT5716, *L. casei* B255, *L. reuteri* DSM 17509, *L. plantarum* LP299v, and *L. acidophilus* – significantly reduce pro-inflammatory cytokines (TNF-α, IL-12, IL-6, IL-1β, IL-17, and interferon-gamma) while elevating anti-inflammatory IL-10 and Treg cells^[^22^]^. In contrast, Aparicio-Pascual *et al* reported no significant reduction in pro-inflammatory cytokines in their meta-analysis^[^23^]^. However, our study observed decreased IL-1β, IL-2, and IL-6 levels in the F + P3 treatment group. This discrepancy highlights the importance of strain-specific effects and experimental design in probiotic research. The observed dose-dependent anti-inflammatory response indicates promising therapeutic potential and underscores the need for further dose-ranging clinical trials in both acute and chronic inflammatory conditions. However, comparative data on the efficacy of varying doses of the same probiotic across specific health conditions and populations are often lacking, making it difficult to establish clear dose–effect relationships for most probiotics.

Also, the anti-inflammatory effects of probiotics could be influenced by their origin. *L. plantarum* and *Bifidobacterium longum* strains inhibit the nuclear factor kappa B (NF-κB) inflammatory pathway, reducing inflammation^[^24^]^. *L. casei* strains have been shown to increase anti-inflammatory cytokines (IL-10 and TGF-β) and suppress pro-inflammatory cytokines (IL-1β, IL-2, IL-6, IL-12, and IL-17) in rheumatoid arthritis models^[^25^]^. Additionally, systematic reviews and meta-analyses have summarized randomized controlled trials involving various probiotic species such as *L. casei, Bacillus coagulans, L. acidophilus, Lactococcus lactis, Bifidobacterium lactis*, and *Bifidobacterium bifidum* in inflammatory arthritis and other autoimmune diseases^[^26,27^]^. These studies report that different probiotic strains can reduce inflammatory markers like C-reactive protein, TNF-α, and IL-6, and improve clinical symptoms, although results vary depending on the strain and disease context. In this study, we utilized *L. plantarum* NIMBB003. Considering acetaminophen’s well-known anti-inflammatory properties^[^28,29^]^, we observed that this probiotic NIMBB003 strain exhibited effects in the same direction as anti-inflammatory and analgesic properties.

Studies on probiotics show different administration times. A specific clinical study administering probiotics to healthy women for 4 weeks found no significant changes in gut microbiota composition during or shortly after probiotic intake, suggesting that short-term administration may not always alter microbial diversity^[^30–32^]^. Systematic reviews show that probiotic supplementation periods in clinical trials typically range from about 4 to 16 weeks, with a median duration of around 60 days^[^33^]^. This duration is often chosen to balance observing effects while maintaining participant compliance^[^34,35^]^. The results of this study were observed after 2 weeks of administration. We suggest that the effects of the duration of administration of this probiotic be investigated in clinical studies.

The difference in administration duration between the probiotic and acetaminophen reflects their fundamentally different mechanisms of action. Acetaminophen is a fast-acting analgesic that exerts its effect primarily through central inhibition of cyclooxygenase pathways and modulation of serotonergic systems. Because of its rapid absorption and relatively short half-life, a single dose administered 20 minutes prior to the formalin test is sufficient to produce measurable acute analgesic effects during the nociceptive phases.

In contrast, probiotics do not act as immediate pharmacological analgesics. Their effects are mediated through gradual modulation of the gut microbiota, enhancement of intestinal barrier integrity, regulation of immune responses, and interaction with the gut–brain axis. These biological processes require sustained administration to allow colonization, microbiota remodeling, and downstream immunomodulatory and neurochemical changes. Therefore, a 14-day pretreatment period is necessary to establish a stable physiological effect before a nociceptive challenge^[^2,36–38^]^.

Therefore, a 14-day pretreatment protocol is necessary to allow sufficient microbiota and immune remodeling so that the probiotic’s maximal effect is present at the time of FI. In contrast, acetaminophen acts acutely and does not require such a build-up phase. The design goal was to synchronize the maximum effect at the moment of testing, not to equalize the duration of treatment.

## Conclusion

The administration of *L. plantarum* NIMBB003 effectively reduced acute inflammatory pain in a dose-responsive fashion. Furthermore, the maximum tested dose (10^11^ CFU) demonstrated significant analgesic activity in both early and late phases of the formalin-induced pain model, and its efficacy was in the same direction as that of the reference analgesic, acetaminophen. The suppression of inflammatory pathways may have played a role in the observed analgesic effect. Pretreatment with the high-dose probiotic markedly reduced the formalin-induced elevation of pro-inflammatory cytokines, specifically IL-1β, IL-2, and IL-6. This anti-inflammatory effect was similar to that achieved by acetaminophen, which is indicative of the probiotic’s potential as an immunomodulatory treatment. Although lower doses of the probiotic were ineffective, further studies are needed to demonstrate a clear dose–response relationship and determine the optimal dose to achieve therapeutic benefits after administration of *L. plantarum* NIMBB003.

### Limitation and suggestion

In this study, we directly compared long-term probiotic pretreatment with a single acute dose of acetaminophen to evaluate their relative effects under equivalent dosing conditions. We agree that future studies should examine higher doses of both acetaminophen and *Lactiplantibacillus plantarum* NIMBB003 to better characterize dose–response relationships. To our knowledge, this is the first study to directly compare acetaminophen with *L. plantarum* NIMBB003. Although an initial sample size of *n* = 3 may be acceptable for exploratory purposes, increasing the sample size to at least *n* = 6 or more would provide greater statistical power and improve the robustness and reliability of the findings in future investigations.

We also recommend that subsequent studies incorporate mechanistic endpoints to establish a clearer causal link between probiotic exposure and pain modulation. Such approaches may include the assessment of fecal colonization (e.g., 16S rRNA sequencing or qPCR targeting the administered strain), evaluation of intestinal permeability markers, or measurement of inflammatory markers in the spinal cord. Finally, further research – particularly well-designed clinical trials in human populations – is necessary to determine the optimal therapeutic dose, evaluate long-term safety, and clarify the precise mechanisms underlying the observed analgesic and anti-inflammatory effects in both early- and late-phase pain contexts.

## Data Availability

This study’s raw results and data are the responsibility of the corresponding authors and will be provided upon receiving a valid reason.

## Research registration unique identifying number (UIN)

Not applicable. This is an animal experimental study and was not prospectively registered.

## Author agreement

This paper is submitted with the agreement of all its co-authors, and the author list has been approved by all authors.
